# Supplementary figures and images for: Are most published research findings false in a continuous universe?
Source: PLoS One. 2022 Dec 20;17(12):e0277935. doi: 10.1371/journal.pone.0277935 (PMC9767354; doi:10.1371/journal.pone.0277935)

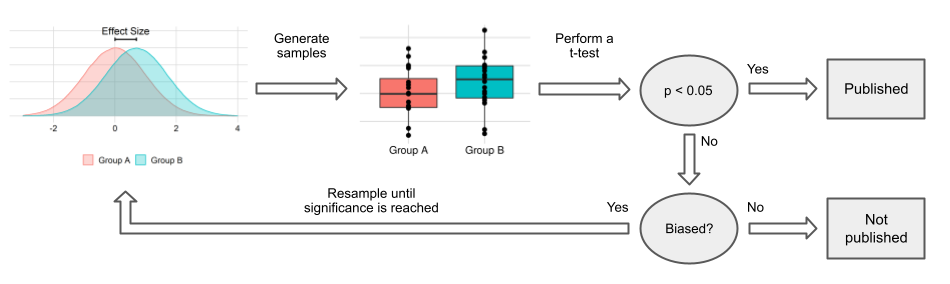

Supplement: S1 Fig — Given an effect size drawn from the distributions described in Fig 1, samples are generated for two groups, from normal probability distributions whose means differ by the effect size. A t-test is performed comparing these two samples and a p-value is obtained. If that p-value is significant at the given alpha level, the result is published. If it is not, there is a chance that the result will be biased, with a probability given by the bias parameter. If it is, new samples will be generated until significance is reached and the result is published. Otherwise, the result is not published. (TIF) [file pone.0277935.s001.tif]

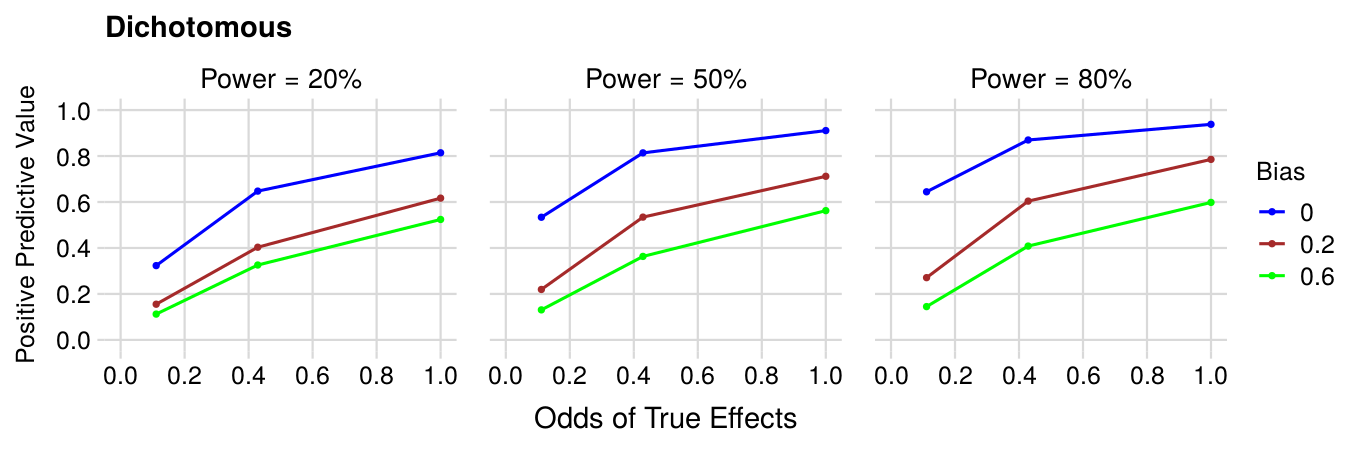

Supplement: S2 Fig — These are the same results shown in Fig 2A(capped at an odds ratio of 1), and are meant to be more directly comparable to the figures in Ioannidis [1], which specify prevalence using odds instead of percentages. Each point corresponds to a simulation of 5,000 published findings. True effects are defined as those above the minimum of interest (i.e. Cohen’s d> 0.5 in all simulations). In each panel, graphs correspond to 20%, 50% and 80% power from left to right. Blue, red and green curves correspond to bias of 0, 0.2 and 0.6, respectively. (TIF) [file pone.0277935.s002.tif]

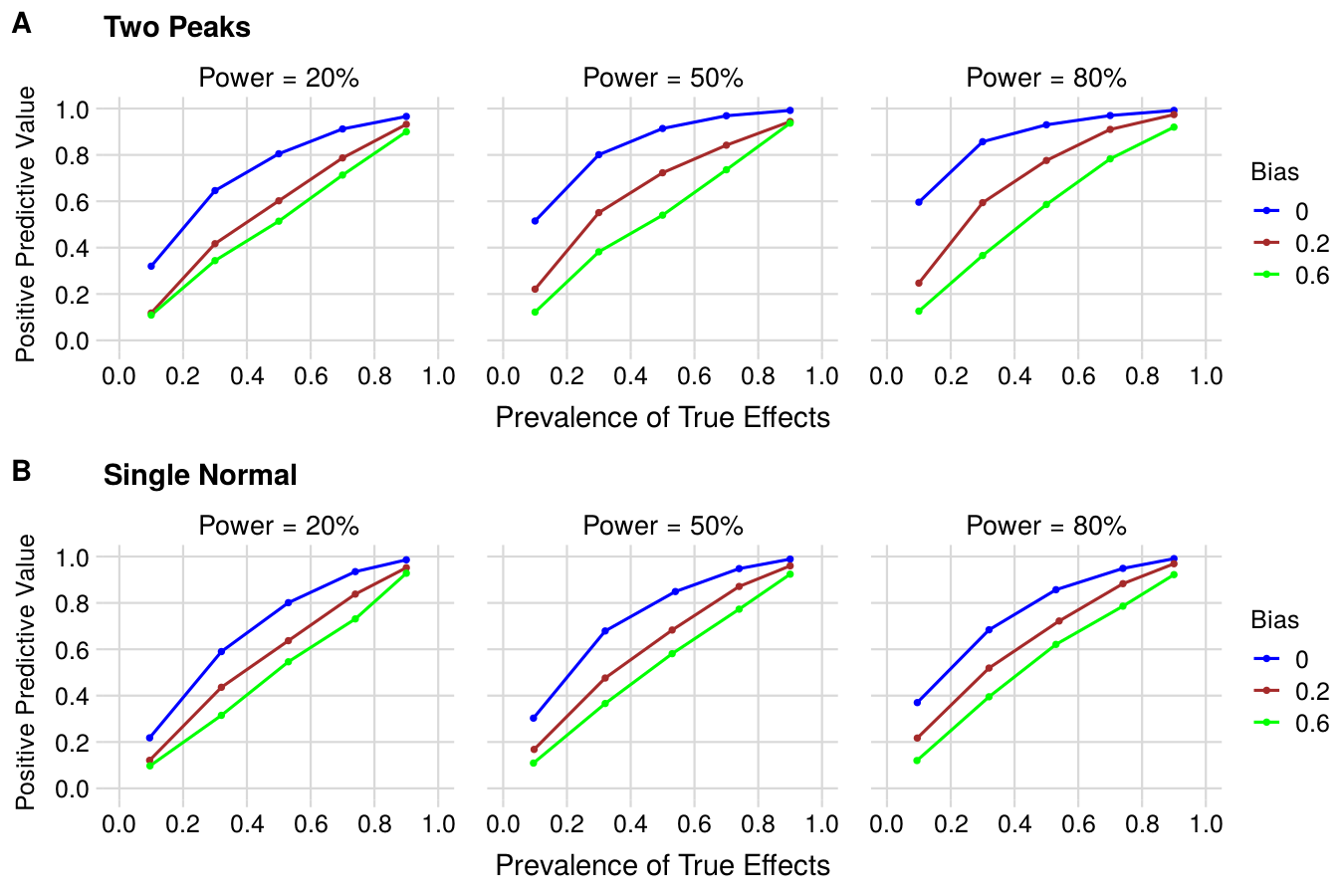

Supplement: S3 Fig — These results are similar to those presented in Fig 2, except that the decision to publish takes the estimated effect size into account–i.e. results are only published if the estimate was larger than the minimum of interest. Each point corresponds to a simulation with 1,000 published findings. True effects are defined as those above the minimum of interest (i.e. Cohen’s d> 0.5 for these simulations). In each panel, graphs correspond to 20%, 50% and 80% power from left to right. Blue, red and green curves correspond to bias of 0, 0.2 and 0.6, respectively. Prevalence ranges vary among scenarios, as for some distributions some prevalences cannot be achieved without changing other parameters. (TIF) [file pone.0277935.s003.tif]

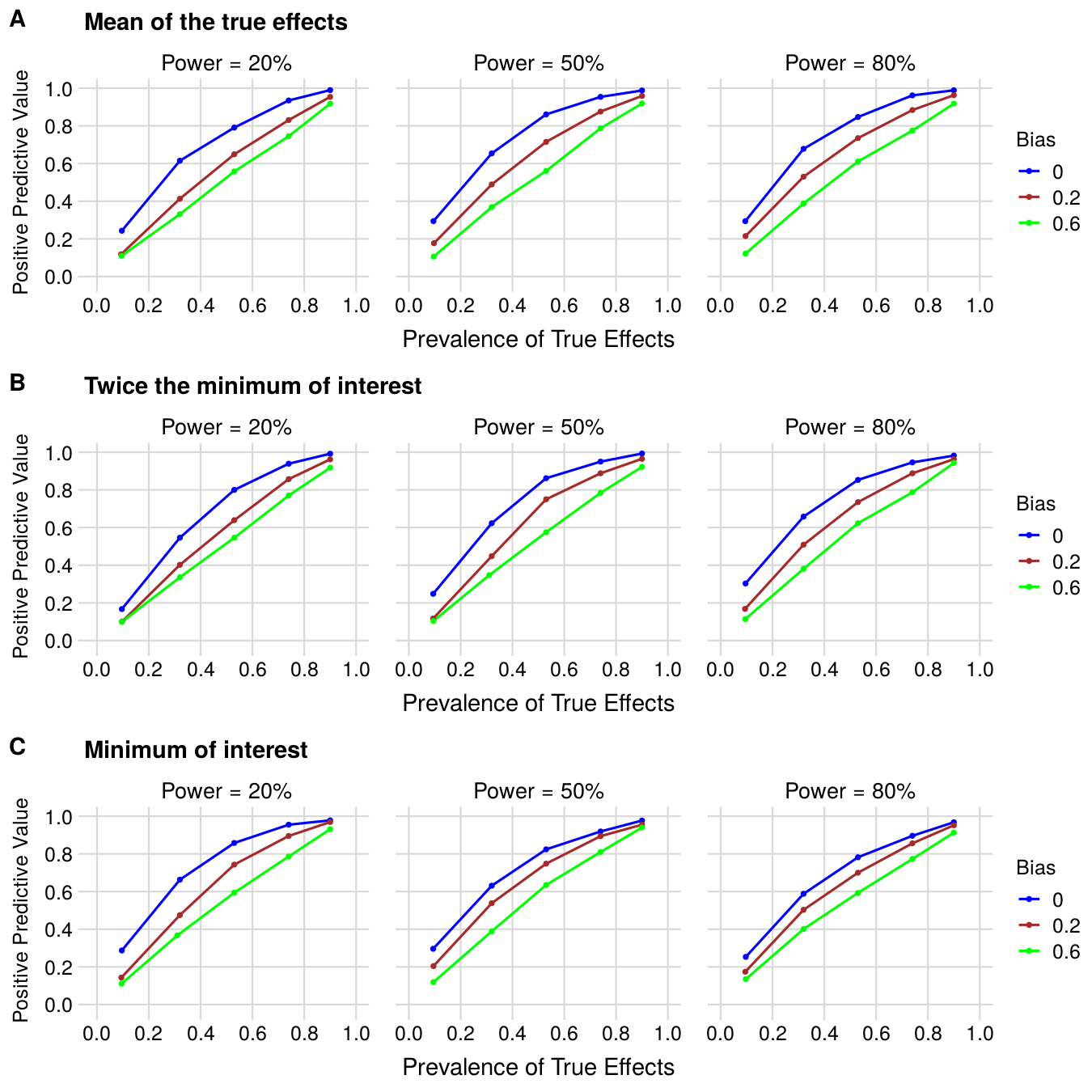

Supplement: S4 Fig — Each point corresponds to a simulation with 1,000 published findings. In each panel, graphs correspond to 20%, 50% and 80% power from left to right. Blue, red and green curves correspond to bias of 0, 0.2 and 0.6, respectively. Prevalence ranges vary among scenarios, as for some distributions some prevalences cannot be achieved without changing other parameters. (TIF) [file pone.0277935.s004.tif]

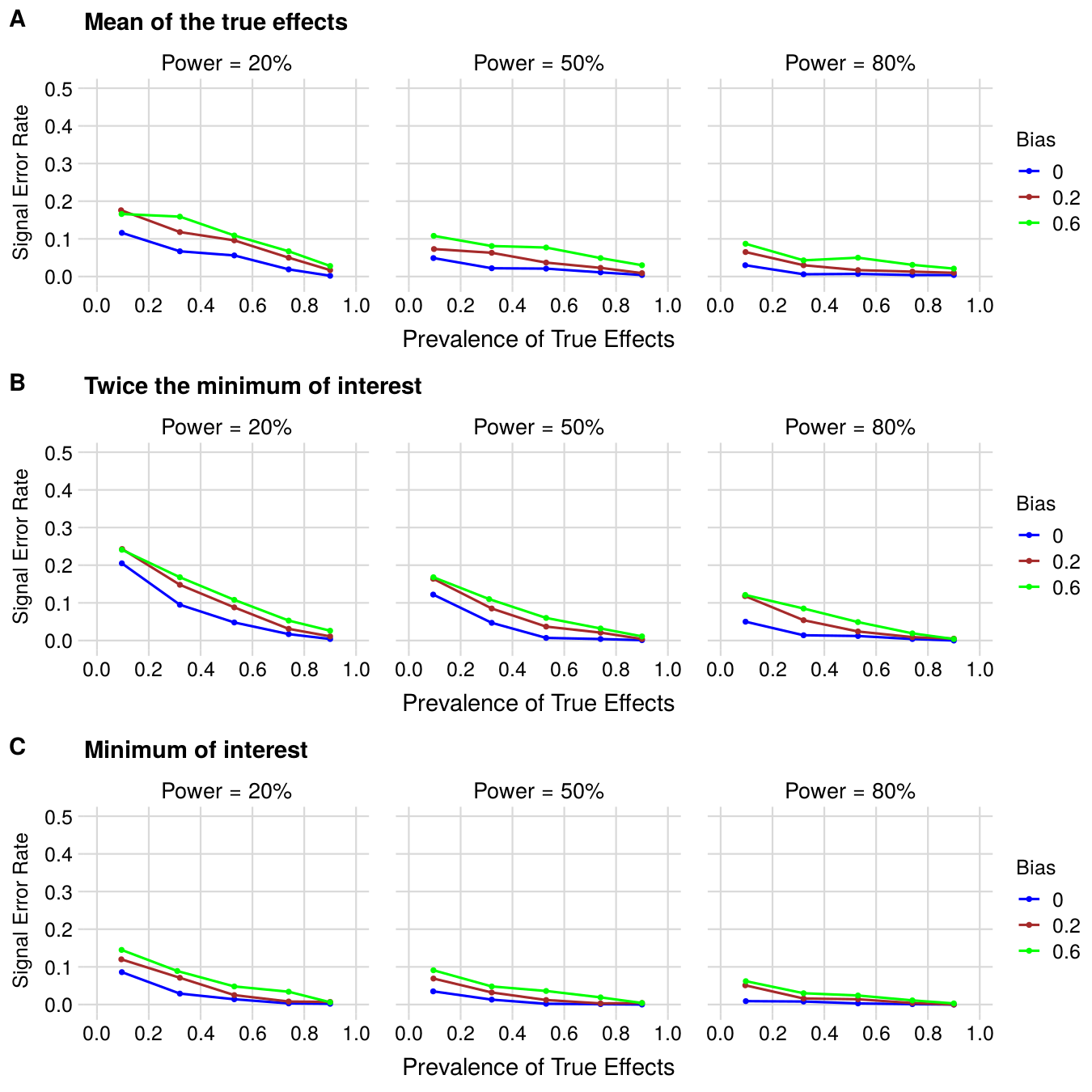

Supplement: S5 Fig — Plots show histograms of the distribution of p-values for published (i.e. significant) results for 1,000 findings, in different scenarios. Top panels show results for the Two Peaks model, and bottom ones for the Single Normal model. Left panels show results for a high-bias, low-power, low-prevalence scenario, while right ones have no bias, high power and high prevalence. Specific parameters are (A) Two Peaks, bias = 0.6, power = 20%, prevalence ~ 0.1,PPV ~ 11%(B)Two Peaks, bias = 0, power = 80%, prevalence ~ 0.9, PPV ~ 12%, (C)Single Normal, bias = 0.6, power = 0.2, prevalence ~ 0.1, PPV ~ 99% and (D)Single Normal, bias = 0, power = 0.8, prevalence ~ 0.9, PPV ~ 99%. (TIF) [file pone.0277935.s005.tif]

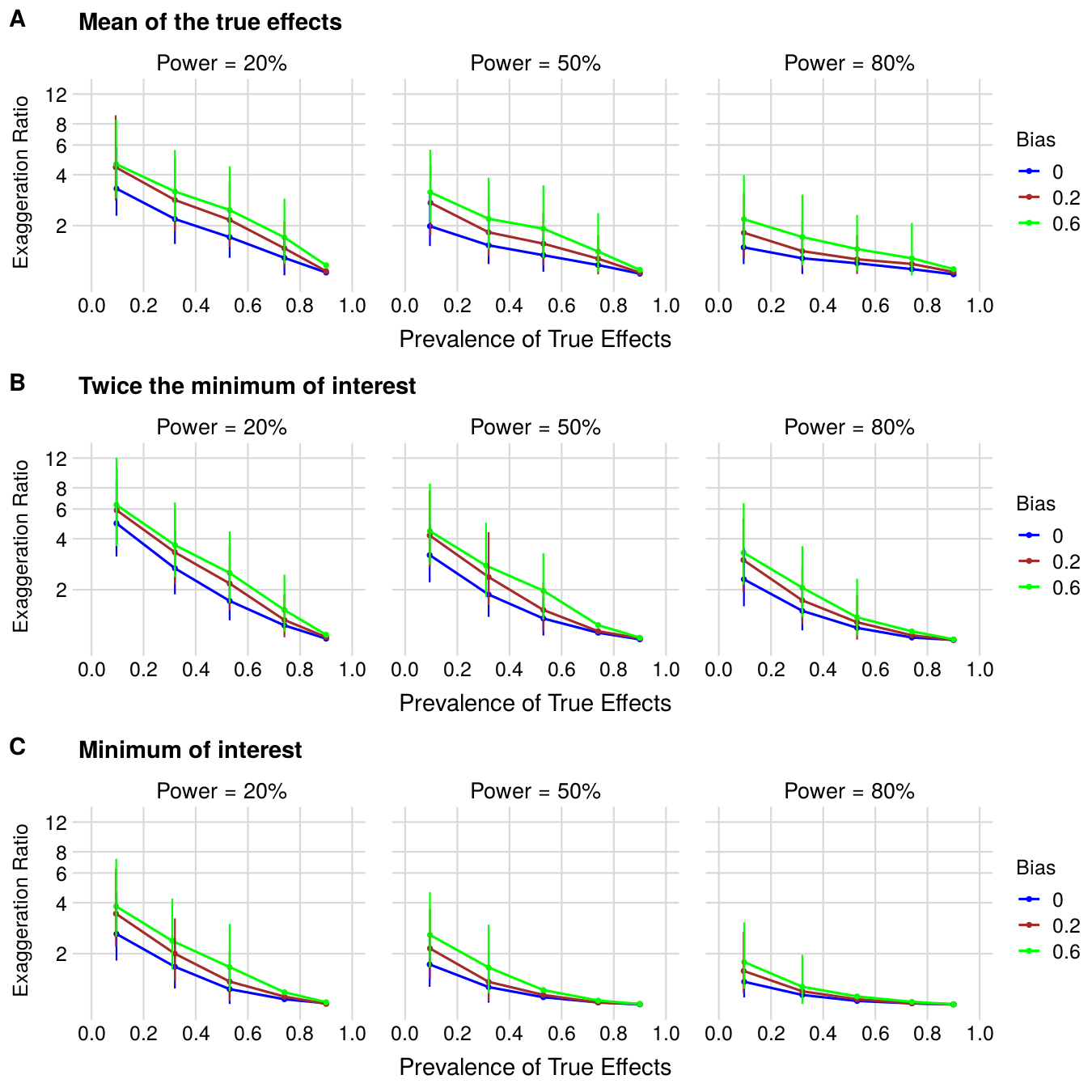

Supplement: S6 Fig — In (A), sample size is calculated to detect the mean of the true effect sizes above the minimum of interest (i.e. Cohen’s d > 0.5). This is the method used in the main simulations. As prevalence in the Single Normal model is increased by increasing the SD, the mean effect size above the minimum is larger as prevalence increases, leading simulations to be underpowered compared to other distributions at high prevalences. In (B), sample size is calculated to detect an effect equal to twice the minimum of interest (i.e. Cohen’s d = 1). In (C), it is calculated to detect an effect equal to the minimum of interest (i.e. Cohen’s d = 0.5). Each point corresponds to a simulation with 1,000 published findings. True effects are defined as those above the minimum of interest. In each panel, graphs correspond to 20%, 50% and 80% power from left to right. Blue, red and green curves correspond to bias of 0, 0.2 and 0.6, respectively. (TIF) [file pone.0277935.s006.tif]

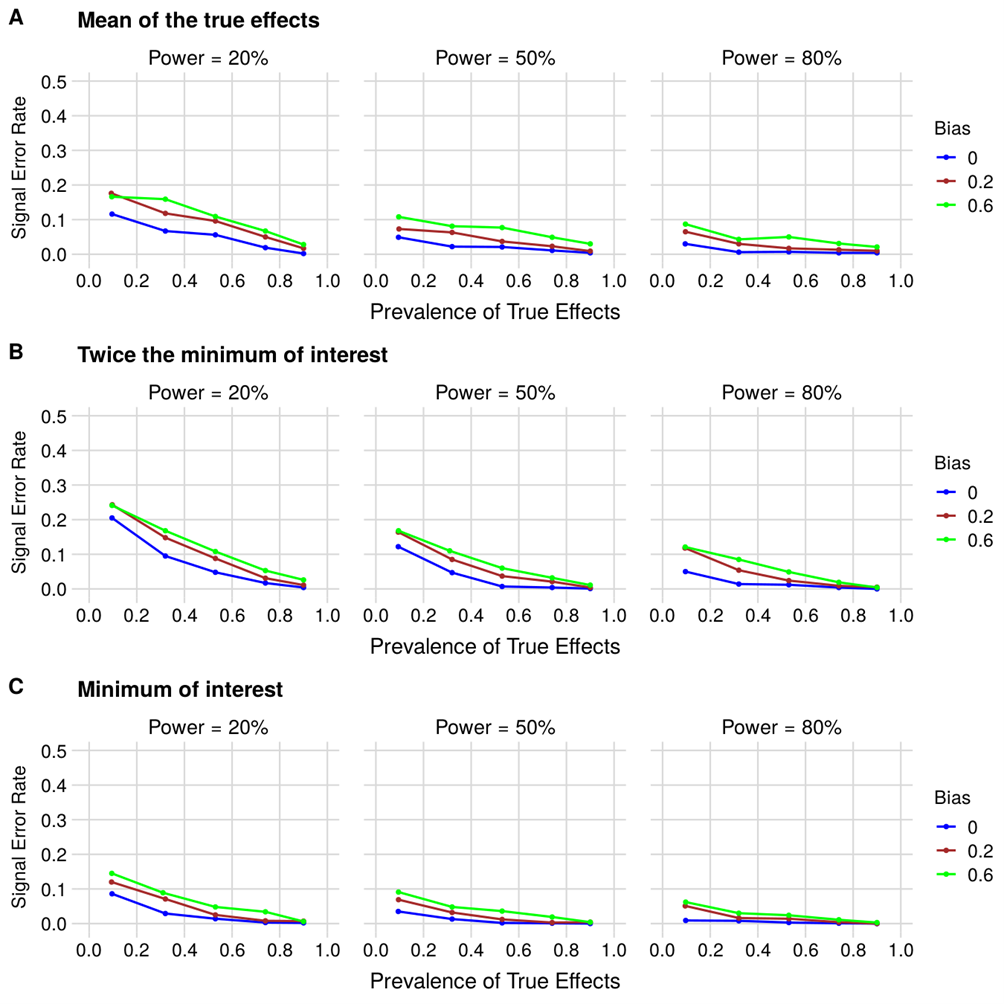

Supplement: S7 Fig — In (A), sample size is calculated to detect the mean of the true effect sizes above the minimum of interest (i.e. Cohen’s d > 0.5). This is the method used in the main simulations. As prevalence in the Single Normal model is increased by increasing the SD, the mean effect size above the minimum is larger as prevalence increases, leading simulations to be underpowered compared to other distributions at high prevalences. In (B), sample size is calculated to detect an effect equal to twice the minimum of interest (i.e. Cohen’s d = 1). In (C), it is calculated to detect an effect equal to the minimum of interest (i.e. Cohen’s d = 0.5). Each point corresponds to a simulation with 1,000 published findings. True effects are defined asthose above the minimum of interest. In each panel, graphs correspond to 20%, 50% and 80% power from left to right. Blue, red and green curves correspond to bias of 0, 0.2 and 0.6, respectively. (TIF) [file pone.0277935.s007.tif]

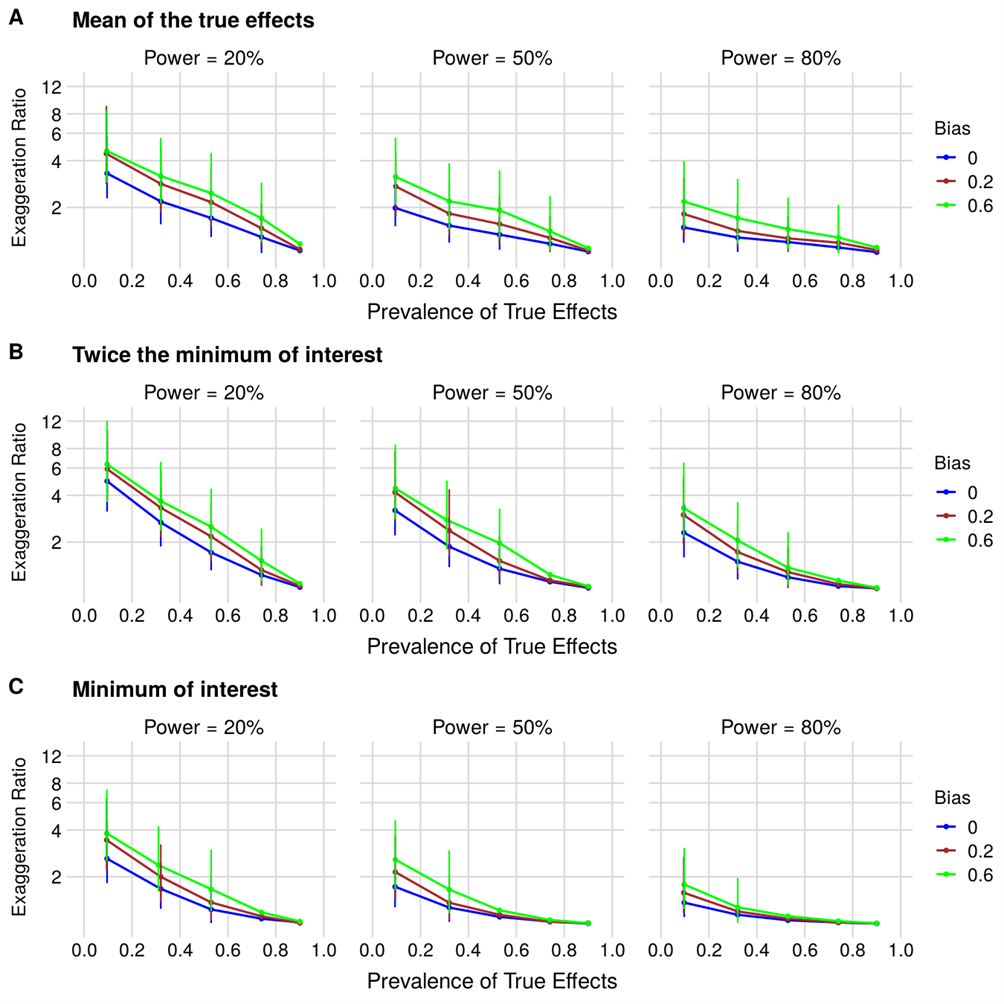

Supplement: S8 Fig — In (A), sample size is calculated to detect the mean of the true effect sizes above the minimum of interest (i.e. Cohen’s d > 0.5). This is the method used in the main simulations. As prevalence in the Single Normal model is increased by increasing the SD, the mean effect size above the minimum is larger as prevalence increases, leading simulations to be underpowered compared to other distributions at high prevalences. In (B), sample size is calculated to detect an effect equal to twice the minimum of interest (i.e. Cohen’s d = 1). In (C), it is calculated to detect an effect equal to the minimum of interest (i.e. Cohen’s d = 0.5). Each point corresponds to a simulation with 1,000 published findings. True effects are defined as those above the minimum of interest. In each panel, graphs correspond to 20%, 50% and 80% power from left to right. Blue, red and green curves correspond to bias of 0, 0.2 and 0.6, respectively. (TIF) [file pone.0277935.s008.tif]
